# Serum Levels of Brain-Derived Neurotrophic Factor and Insulin-Like Growth Factor 1 Are Associated With Autonomic Dysfunction and Impaired Cerebral Autoregulation in Patients With Epilepsy

**DOI:** 10.3389/fneur.2018.00969

**Published:** 2018-11-20

**Authors:** Shu-Fang Chen, Shuo-Bin Jou, Nai-Ching Chen, Hung-Yi Chuang, Chi-Ren Huang, Meng-Han Tsai, Teng-Yeow Tan, Wan-Chen Tsai, Chiung-Chih Chang, Yao-Chung Chuang

**Affiliations:** ^1^Department of Neurology, Kaohsiung Chang Gung Memorial Hospital, Chang Gung University College of Medicine, Kaohsiung, Taiwan; ^2^Department of Neurology, Mackay Memorial Hospital and Mackay Medical College, Taipei, Taiwan; ^3^Department of Occupational and Environmental Medicine, Kaohsiung Medical University Hospital and School of Public Health, Kaohsiung Medical University, Kaohsiung, Taiwan; ^4^Institute for Translational Research in Biomedicine, Kaohsiung Chang Gung Memorial Hospital, Kaohsiung, Taiwan; ^5^Department of Neurology, Faculty of Medicine, College of Medicine, Kaohsiung Medical University, Kaohsiung, Taiwan; ^6^Department of Biological Science, National Sun Yat-sen University, Kaohsiung, Taiwan

**Keywords:** epilepsy, autonomic nervous system dysfunction, cerebral autoregulation, brain-derived neurotrophic factor, insulin-like growth factor 1

## Abstract

**Background:** Brain-derived neurotrophic factor (BDNF) and insulin-like growth factor 1 (IGF-1) may regulate the autonomic nervous system (ANS) in epilepsy. The present study investigated the role of IGF-1 and BDNF in the regulation of autonomic functions and cerebral autoregulation in patients with epilepsy.

**Methods:** A total of 57 patients with focal epilepsy and 35 healthy controls were evaluated and their sudomotor, cardiovagal, and adrenergic functions were assessed using a battery of ANS function tests, including the deep breathing, Valsalva maneuver, head-up tilting, and Q-sweat tests. Cerebral autoregulation was measured by transcranial doppler during the breath-holding test and the Valsalva maneuver. Interictal serum levels of BDNF and IGF-1 were measured with enzyme-linked immunosorbent assay kits.

**Results:** During interictal period, reduced serum levels of BDNF and IGF-1, impaired autonomic functions, and decreased cerebral autoregulation were noted in patients with epilepsy compared with healthy controls. Reduced serum levels of BDNF correlated with age, adrenergic and sudomotor function, overall autonomic dysfunction, and the autoregulation index calculated in Phase II of the Valsalva maneuver, and showed associations with focal to bilateral tonic-clonic seizures. Reduced serum levels of IGF-1 were found to correlate with age and cardiovagal function, a parameter of cerebral autoregulation (the breath-hold index). Patients with a longer history of epilepsy, higher seizure frequency, and temporal lobe epilepsy had lower serum levels of IGF-1.

**Conclusions:** Long-term epilepsy and severe epilepsy, particularly temporal lobe epilepsy, may perturb BDNF and IGF-1 signaling in the central autonomic system, contributing to the autonomic dysfunction and impaired cerebral autoregulation observed in patients with focal epilepsy.

## Introduction

The interaction between epileptic seizures and the autonomic nervous system (ANS) is very complex (1). Theoretically, epileptic seizures, which involve the propagation of abnormal neuronal electrical activities, may interact with the ANS through central autonomic centers and contribute to the regulation of autonomic activity (1, 2). In addition, epilepsy can present with autonomic dysfunction during seizures (3). Patients with epilepsy may experience prolonged changes in ANS functions, which can influence ANS target organs, such as the heart, stomach, intestines, and lungs, resulting in clinical presentations of ANS dysfunction (1, 2). Cortical autonomic dysfunction may lead to various cardiac dysrhythmias, including tachycardia, bradycardia, and QT prolongation during ictal and interictal states of epilepsy (4, 5). Importantly, the involvement of autonomic functions in patients with epilepsy, in particular, effects on cardiorespiratory function, may contribute to sudden unexpected death in epilepsy (SUDEP) (6–8). Manifestations of autonomic changes in cardiovascular (2, 4, 8) and respiratory (9) function, and the dysfunction of sympathetic-mediated cerebral autoregulation (10) have been suggested as possible mechanisms underlying SUDEP.

Neurotrophic factors play a crucial role in regulating neurons in the ANS, and in the formation of synaptic connections with peripheral targets in the cardiovascular, digestive, and other organ systems during development. They also influence the function of the ANS in adults (11). Neurotrophic factors, including brain-derived neurotrophic factor (BDNF), nerve growth factor, insulin-like growth factor (IGF), and ciliary neurotrophic factor may regulate ANS functions (11). Recent studies have found that BDNF and IGF-1 can modify the functions of the central nervous system (CNS) that are involved in neurological and psychiatric diseases, including epilepsy (12–14). Evidence from animal studies (15–17) has shown that BDNF and IGF-1 also play a partial role in the pathophysiology and epileptogenesis of epilepsy. Epileptic seizures may change the expression levels of both BDNF mRNA and protein in hippocampal neurons (15, 17). Administration of IGF-I decreased seizure severity and hippocampal neurogenesis, which ameliorated hippocampal neurodegeneration and protected against cognitive deficits, in an experimental animal model of temporal lobe epilepsy (16). However, the role of BDNF and IGF-1 in epilepsy in humans has been explored only to a limited extent. Our recent study (18) found that lower serum levels of BDNF may indicate a longer duration of epilepsy, impaired white matter integrity, and poor cognitive function in patients with chronic temporal epilepsy.

Although both seizure activity and neurotrophic factors can modulate ANS function, their role in the regulation of autonomic function and cerebral autoregulation in patients with epilepsy is unclear. In the present study, we tested the hypothesis that the neurotrophic factors IGF-1 and BDNF play a crucial role in the regulation of autonomic functions and cerebral autoregulation in patients with epilepsy.

## Materials and methods

This was a prospective case-control study conducted at Kaohsiung Chang Gung Memorial Hospital. Kaohsiung Chang Gung Memorial Hospital is a tertiary medical center in Taiwan. The institutional ethics committee approved the study protocol, and informed written consent was obtained from all subjects.

### Subjects

A total of 57 patients (28 males and 29 females) with focal epilepsy (aged between 18 and 65 years) who received treatment for more than 2 years were enrolled in the present study through the Epilepsy Outpatient Clinic of Kaohsiung Chang Gung Memorial Hospital. Thirty-five healthy volunteers (17 males and 18 females) were recruited to serve as normal controls. The average age of the patients with epilepsy was 36.5 ± 10.44 (mean ± standard deviation) years, and 36.3 ± 9.29 years in the control group. All recruited subjects were free of diseases that can affect the ANS, including cerebrovascular diseases, cardiovascular diseases, severe traumatic brain injury, neurodegenerative diseases, polyneuropathies, hypertension, diabetes mellitus, endocrine diseases, and autoimmune diseases. Subjects taking medicines which can affect ANS function were also excluded from the study. All subjects underwent physical and neurological examinations by two neurologists and received evaluations of ANS function in the morning (between 8:00 a.m. and 12:00 noon). Age, gender, body weight, body height, and body mass index (BMI) were recorded. Clinical information for the patients with epilepsy were obtained from clinical records and interventions, including etiology of epilepsy, seizure types, duration of epilepsy, current antiepileptic drug (AED), therapeutic state, frequency of seizures, findings of electroencephalography, and results of neuroimaging studies. The seizure types and semiology were classified according to the 2017 recommendations of the International League Against Epilepsy (ILAE) (19).

### Measurement of serum BDNF and IGF-1 levels

Blood samples for BDNF and IGF-1 analysis were obtained from patients and controls between 8:00 and 10:00 a.m., following overnight fasting, in the interictal state. Serum samples were separated by centrifugation (3,000 rpm for 10 min at 4°C) and were collected in anticoagulant-free tubes containing a clotting activator and kept on ice for 1 h at a temperature of 4°C. Serum levels of BDNF and IGF-1 were measured by using enzyme-linked immunosorbent assay kits (BDNF: BDNF E_max_^®^Promega Corporation, Madison, WI, USA; IGF−1: AssayMax^TM^ human IGF −1 ELISA kit, ASSAYPRO, St. Charles, MO, USA) (18). The degree of enzymatic turnover of the substrate was determined by dual wavelength absorbance measurements at 450nm, using a multiscan spectrum reader(Thermo Scientific, Miami, FL, USA)according to the manufacturer′s instruction.

### Evaluation of ANS functions

A standardized ANS evaluation of cardiovagal, adrenergic, and sudomotor functions was performed using a battery of tests (20, 21). The test battery consisted of a deep breathing test, Valsalva maneuver, head-up tilt test, and Q-sweat test. Cardiovagal function was evaluated by testing the heart rate (HR) response to deep breathing at a defined rate and to the Valsalva maneuver (22). Adrenergic function was evaluated by blood pressure (BP) and HR responses to the Valsalva maneuver and to a head-up tilt (22). Sudomotor function was evaluated with a quantitative sudomotor axon reflex test (QSART) using the Q-sweat test (22, 23). HR was recorded with a standard three-lead electrocardiogram (ECG; Ivy Biomedical, model 101; Branford, CT, USA) and arterial BP was measured at the finger using beat-to-beat photoplethysmographic recordings (Finapres BP monitor 2300, Ohmeda; Englewood, CO, USA). ECG and BP were recorded during normal breathing following a 15-min rest period in the supine position. Sweat output was recorded with a Q-Sweat device (WR Medical Electronics Co., Stillwater, MN, USA).

#### Deep breathing test

Subjects were equipped with a beat-to-beat BP device, ECG, and chest bellows, in a supine position. After a 1-min baseline recording, subjects were evaluated for HR range during six deep breaths in 1 min. The procedure was repeated three times, each time following a 2-min resting period. The amplitude of the beat to beat variation with respiration was measured and calculated with TestWorks software (WR Medical Electronics Co., Maplewood, MN, USA).

#### Valsalva maneuver

The Valsalva maneuver was repeated until two responses of similar beat-to-to beat BP and HR were obtained. After a 1-min baseline recording, subjects underwent three repetitions of the Valsalva maneuver test. The Valsalva ratio (VR) was derived from the maximum HR generated by the Valsalva maneuver divided by the lowest HR occurring within 30 s of the peak HR. During the Valsalva maneuver, beat-to-beat BP was continuously monitored by the Finapres monitor and analyzed by the TestWorks software.

#### Head-up tilt test

After a 5-min baseline recording in the supine position, the table was tilted at an angle of 70 degrees. Beat-to-beat HR, systolic, diastolic, and mean BP were continuously recorded by Finapres and ECG. Manual BP was obtained 1 min before the head-up tilt test, and at 1, 2, 3 and 5 min during the head-up tilt, and 1 min post-head-up tilt.

#### Quantitative sudomotor axon reflex test (Q-sweat test)

The protocol for the Q-sweat test, with respect to recording sites, skin preparation, and testing procedure, was carried out as previously described (23).

#### The modified composite autonomic scoring scale

The composite autonomic scoring scale (CASS) by the Mayo-QSART (21) was modified (mCASS) and developed in our laboratory (we used the Q-sweat test instead of a thermoregulatory sweat test for the purpose of the study), and served as an indicator of general autonomic function (21, 23). The scheme allotted 4 points for adrenergic failure and 3 points each for sudomotor and cardiovagal failure (24). The results of the battery of tests for autonomic function were corrected for the confounding effects of age and sex. The tests were graded semiquantitatively from 0 (no deficit) to 10 (maximal deficit). The mCASS consisted of three subscores: sudomotor (0–3); cardiovagal (0–3); and adrenergic (0–4).

### Measurement of cerebral autoregulation

Cerebral hemodynamic function was evaluated by transcranial doppler (TCD) sonography using a headband (Doppler Box, DWL; Compumedics, Charlotte, NC, USA) in a temperature- and humidity-controlled environment between 9:00 a.m. and 2:00 p.m. on the same day as the other examinations. Mean cerebral blood flow velocity (CBFV) at the bilateral proximal middle cerebral artery (MCA) was measured using TCD (Multidop XL™, DWL, Sipplingen, Germany). The MCA was insonated through the temporal window approximately 1 cm above the zygomatic arch at a depth of 35–55 mm, using pulsed 2 MHz-Doppler probes. The MCA was continuously monitored during breath holding and the Valsalva maneuver.

After a 5 min resting period, a value of the continuous mean velocity monitored by TCD over 30 s was collected to obtain baseline data. After normal inspiration, subjects held their breath for 30 s and the CBFV of the final 3 s of the breath holding period were recorded. The procedure was repeated three times, each time after a 2-min resting period. The Breath Holding Index (BHI) was calculated as the percentage increase in CBFV during breath-holding, divided by the duration for which the subject held their breath (25).

The Valsalva maneuver and the TCD monitoring were performed simultaneously. The BP, HR, and CBFV were measured continuously with Beatscope Easy software (Finapres Medical Systems B.V., Enschede, Netherlands). An autoregulatory index for Phase II (ASI) was calculated as: ASI = (ΔCBFV/CBFV_II_) – (ΔBP/BP_II_) × 100%. CBFV_II_ and BP_II_ were measured at the beginning of the CBFV recovery slop during phase II (26, 27). The differences ΔCBFV and ΔBP were calculated for the subsequent 3 s (26, 27).

### Statistical analysis

All data were displayed as mean ± standard deviation for continuous variables. The Mann–Whitney *U*-test was used to compare variables between groups. Differences in adrenergic, cardiovagal, sudomotor, and mCASS subscores between patients with epilepsy and controls were calculated by the Fisher's exact test. Differences in age, sex, BMI, serum levels of BDNF and IGF-1 and parameters of cerebral autoregulation (BHI and ASI), between patients with epilepsy and controls were calculated separately by the Mann–Whitney *U*-test. Spearman's rank correlation analysis was used to explore the relationship between serum levels of BDNF and IGF-1, and autonomic scores and parameters of cerebral autoregulation. The correlation between serum levels of BDNF and IGF-1 and seizure semiology were analyzed by Spearman's correlation analysis. The difference of the types of seizure semiology between serum levels of BDNF and IGF-1 were calculated by the Mann–Whitney *U-*test. A *p* < 0.05 was taken to indicate statistical significance. All statistical analysis was conducted using the Statistical Package for Social Sciences software package (SPSS Inc., Chicago, IL, USA).

## Results

### Characteristics and demographic data

With respect to gender and age, there were no significant differences between patients and control subjects. Base on statistical analysis, BMI in the patients with epilepsy was significantly higher than in controls (24.6 ± 5.02 vs. 22.3 ± 2.84; *p* = 0.033). Clinical characteristics and demographic data of the 57 patients with epilepsy are shown in Table 1.

**Table 1:** Clinical characteristics and demographic data for the patients with epilepsy.

Age (years)	36.5 ± 10.44
Male	37.4 ± 10.85
Female	35.5 ± 10.14
Sex (male: female)	28: 29
Age at onset (years)	17.6 ± 10.49
Duration of epilepsy (years)	19.6 ± 10.37
Seizure frequency (per month)	2.7 ± 6.05
Seizure types, *n* (%)
Focal seizure alone	20 (35.1%)
Focal to bilateral tonic-clonic	37 (64.9%)
Focus of epileptogenesis, *n* (%)
Temporal lobe epilepsy	32 (56.1%)
Extra-temporal lobe epilepsy	25 (43.9%)
Seizure control, *n* (%)
Refractory epilepsy	44 (77.2%)
Seizure free^*^	13 (22.8%)
AED numbers, *n* (%)
Single	11 (19.3%)
Multiple	46 (80.7%)

Values are expressed as mean ± standard deviation.

* *Defined as seizure free for more than 12 months under current AED therapy. AED, antiepileptic drug*.

### Impairment of autonomic function and cerebral autoregulation in patients with epilepsy

Results of the autonomic scores (adrenergic, cardiovagal, sudomotor, and mCASS) and the parameters of cerebral autoregulation (BHI and ASI) in the patients with epilepsy and the controls are shown in Table 2. The autonomic functional scores, including those for adrenergic, cardiovagal, and sudomotor function, and the mCASS, were significantly higher in the patient group than in the controls. Additionally, the tested parameters of cerebral autoregulation (BHI and ASI) were significantly lower in the patients with epilepsy than in the control group.

**Table 2:** Comparison of the modalities in autonomic functions and cerebral autoregulation in patients with epilepsy and the controls.

	**Score**	**Patients with epilepsy (*n* = 57)**	**Controls (*n* = 35)**	***p*-value**
Adrenergic	0	7	35
	1	40	0
	2	10	0	< 0.001^*^
Cardiovagal	0	25	29
	1	20	6
	2	10	0
	3	2	0	0.001^*^
Sudomotor	0	6	31
	1	22	2
	2	11	0
	3	18	2	< 0.001^*^
mCASS	0	0	26
	1	3	6
	2	9	1
	3	16	2
	4	14	0
	5	12	0
	6	2	0
	7	1	0	< 0.001^*^
BHI		0.76 ± 0.298	1.13 ± 0.352	< 0.001#
ASI (%)		−0.17 ± 8.248	7.21 ± 4.798	< 0.001#

Values are expressed the scoring of autonomic function in case numbers and cerebral autoregulation in mean ± standard deviation. mCASS, modified composite autonomic severity score; BHI, Breath-holding index of middle cerebral artery; ASI, a second autoregulation index for phase II in Valsalva Maneuver.

* *Compared difference between patients with epilepsy and the controls by Fisher's exact test. #Compared difference between patients with epilepsy and the controls by the Mann–Whitney U-test*.

### Autonomic function and cerebral autoregulation corelated with serum levels of BDNF and IGF-1 in patients with epilepsy

The serum levels of BDNF and IGF-1 in patients with epilepsy and the controls are shown in Table 3. There was a significant decrease in serum levels of both BDNF and IGF-1 in patients with epilepsy. Based on the correlation analysis (Table 4) of all enrolled subjects, serum levels of BDNF were significantly correlated with age, adrenergic and sudomotor function, and mCASS. Moreover, age and cardiovagal function were significantly correlated with serum levels of IGF-1. ASI, one parameter of cerebral autoregulation, showed a significant correlation with serum levels of BDNF, while BHI was significantly correlated with serum levels of IGF-1.

**Table 3:** Serum levels of BDNF and IGF-1 in patients with epilepsy and controls.

	**Patients with epilepsy (*n* = 57)**	**Controls (*n* = 35)**	***p*-value**
BDNF (ng/mL)	23.31 ± 8.612	29.52 ± 8.394	0.002^*^
IGF-1 (ng/mL)	68.55 ± 40.312	86.66 ± 38.074	0.046^*^

Values are expressed as mean ± standard deviation. BDNF, brain-derived neurotrophic factor; IGF-1, insulin-like growth factor-1.

* *Compared the difference between patients with epilepsy and controls by the Mann–Whitney U-test*.

**Table 4:** Correlation analysis of BDNF and IGF-1 levels with demographic variables, autonomic functions, and cerebral autoregulation parameters in patients with epilepsy and controls.

	**Age**	**Sex**	**BMI**	**Adrenergic**	**Cardiovagal**	**Sudomotor**	**mCASS**	**BHI**	**ASI**
BDNF	−0.247^*^	0.066	0.197	−0.288^*^	−0.083	−0.420^*^	−0.360^*^	0.175	0.256^*^
	(0.018)	(0.530)	(0.060)	(0.005)	(0.434)	(0.001)	(0.001)	(0.138)	(0.035)
IGF-1	−0.411^*^	−0.130	−0.139	0.146	−0.256^*^	−0.085	−0.200	0.261^*^	0.011
	(0.001)	(0.232)	(0.200)	(0.179)	(0.017)	(0.434)	(0.065)	(0.037)	(0.933)

Values are expressed as correlation coefficients (σ) and [significance (p)]. BMI, body mass index; mCASS, modified composite autonomic severity score; BHI, Breath-holding index of middle cerebral artery; ASI, a second autoregulation index for phase II in the Valsalva Maneuver; BDNF, brain-derived neurotrophic factor; IGF-1, insulin-like growth factor-1.

* *Indicates statistical significance (p < 0.05) in the Spearman's rho correlation analysis*.

Results of the correlation analysis between the serum levels of BDNF and IGF-1 and clinical characteristics of the patients with epilepsy are shown in Table 5. With the exception of significantly higher serum levels of BDNF in patients with focal to bilateral tonic-clonic seizures than in those with focal seizures alone (*p* = 0.039), there was no significant correlation between serum levels of BDNF and modalities of seizure semiology. Serum levels of IGF-1 were significantly correlated with the duration of epilepsy, the focus of epileptogenesis, and seizure frequency. Serum levels of IGF-1 in patients with temporal lobe epilepsy were significantly lower than those in patients with extratemporal lobe epilepsy.

**Table 5:** Correlation analysis of BDNF and IGF-1 between the seizure semiology in the patients with epilepsy.

	**BDNF**	**IGF-1**
Age at onset^†^	−0.024 (0.861)	−0.103 (0.445)
Duration of epilepsy^†^	−0.218 (0.103)	−0.363 (0.006)^*^
Seizure frequency^†^	−0.071 (0.598)	−0.303 (0.024)^*^
Seizure types	(*p* = 0.039)#	(*p* = 0.187)
Focal alone (*n* = 20), ng/mL	19.54 ± 9.537	58.68 ± 36.031
Focal with bilateral tonic-clonic (*n* = 37), ng/mL	25.35 ± 7.428	73.89 ± 41.944
Focus of epileptogenesis	(*p* = 0.323)	(*p* = 0.046)#
Temporal epilepsy (*n* = 32), ng/mL	22.03 ± 8.903	59.13 ± 33.159
Extratemporal epilepsy (*n* = 25), ng/mL	24.94 ± 8.106	80.62 ± 45.843
Seizure control	(*p* = 0.470)	(*p* = 0.119)
Refractory epilepsy (*n* = 44), ng/mL	22.94 ± 9.122	64.93 ± 40.224
Seizure free (*n* = 13), ng/mL	24.56 ± 6.760	80.82 ± 39.686
AED numbers	(*p* = 0.225)	(*p* = 0.257)
Single (*n* = 11), ng/mL	20.48 ± 6.706	78.25 ± 40.090
Multiple (*n* = 46), ng/mL	23.98 ± 8.937	66.23 ± 40.456

† Values are expressed in correlation coefficient (σ) and [significance (p)], and the correlation is tested by the Spearman's rho correlation analysis.

* *indicates statistical significance (p < 0.05). Other values of variable are expressed in mean ± standard deviation and compared difference between the different groups is tested by the Mann–Whitney U-test. ^#^Indicates statistical significance (p < 0.05). BDNF, brain-derived neurotrophic factor; IGF-1, insulin-like growth factor-1; AED, antiepileptic drug; n, number of patients*.

## Discussion

The present study confirms that patients with epilepsy frequently experience ANS dysfunction and impaired cerebral autoregulation. These impairments were associated with reduced serum levels of BDNF and IGF-1 during the interictal period. In our study, reduced serum levels of BDNF correlated with age, adrenergic and sudomotor functions, the total autonomic score (mCASS), and cerebral autoregulation (ASI), particularly in patients with focal to bilateral tonic-clonic seizures. Reduced serum levels of IGF-1 correlated with age, cardiovagal function, and cerebral autoregulation (BHI). Meanwhile, patients with a longer duration of epilepsy, higher seizure frequency, and temporal lobe epilepsy had lower serum levels of IGF-1.

BDNF is a member of the neurotrophin family of growth factors (11). It acts on specific neurons of both the central and peripheral nervous systems, affecting synaptic transmission, synaptogenesis, dendridogenesis, and neurogenesis (11, 28, 29). Multiple animal studies (14, 15, 17, 30) have shown that seizure activity is associated with an upregulation of BDNF, at both the mRNA and protein levels, and long-term elevation of BDNF may be a contributory factor in epileptogenesis and the development of refractory epilepsy. In patients with temporal lobe epilepsy (31), an increase in both BDNF mRNA and protein levels was found in surgically resected hippocampus and temporal lobe tissues. While levels of BDNF in the circulation may show variable changes in human patients with epilepsy, our recent study (18) and a previous report (32) both showed significantly decreased levels of serum BDNF in adult patients with epilepsy. The reduced serum level of BDNF is significantly correlated with patients who have focal to bilateral tonic-clonic seizures. Thus, we propose that adult patients with more severe focal motor seizures may have lower serum levels of BDNF.

IGF-1 plays an important role in brain development, maturation, and neuroplasticity (12). In animal models of temporal lobe epilepsy (16, 33), IGF-I promotes neurogenesis and cell survival, which ameliorates seizure-induced hippocampal neurodegeneration and protects against cognitive deficits. However, the effect of IGF-1 on epilepsy remains controversial. In children, low IGF-1 levels are associated with reduced childhood growth, low BMI, low childhood IQ, and autism spectrum disorder (12). In adults, low levels of circulating IGF-1 are correlated with slower mental processing speed and reduced executive functioning (12, 34). In the present study, lower serum levels of IGF-1 were observed in patients with epilepsy, especially in patients with temporal lobe epilepsy. The reduced serum level of IGF-1 was correlated with duration of epilepsy and seizure frequency, which imply that low serum levels of IGF-1 may be related to long-term epilepsy and seizure severity.

Serum concentrations of BDNF have been demonstrated to be stage-dependent, such that increased serum levels of BDNF have been found in early-stage Alzheimer's disease (AD), while decreased serum BDNF levels occur in late-stage AD (35). The decrease in BDNF has been suggested to constitute a lack of trophic support and contribute to the progressive degeneration of specific regions in the affected brains of patients with AD (35). Moreover, in an animal study (36), intracerebroventricular administration of α-ketoisocaproic acid resulted in a reduction in BDNF and nerve growth factor levels in the brain of young rats. In addition, neuronal IGF-1 signaling has been suggested to play a role in the pathogenesis of AD and other neurodegenerative disorders (12, 34). Exogenous IGF-1 has been shown to improve memory in patients with AD and to reduce amyloid-beta levels in the brains of animals (34). In the present study, patients with epilepsy had a long history of epilepsy (19.6 ± 10.37 years) and most of them had refractory epilepsy (77.2%). We propose that reduced serum levels of BDNF and IGF-1 are consistent with the hypothesis that a deficit in these neurotrophic factors may contribute to the structural and functional alterations of the brain underlying the neurodegenerative process related to chronic and severe epilepsy. However, an understanding of the pathophysiological role of reduced serum levels of BDNF and IGF-1 in patients with epilepsy requires further studies in humans.

Autonomic dysfunction is common in a range of neurological disorders, including multiple sclerosis, AD, Parkinson's disease, dementia with Lewy bodies, multiple system atrophy, and epilepsy (1, 11, 37, 38). However, whether perturbed neurotrophic factor signaling is involved in the pathogenesis of ANS dysfunction in patients with epilepsy is still unclear. In an animal model of Rett syndrome (using *MeCP2* mutant mice) (39), systemic treatment with IGF-1 extended the life span of the mice, improved locomotor function, and ameliorated ANS dysfunction. Administration of recombinant human IGF-1 is effective in reversing autonomic dysfunction in Rett syndrome (40). With the exception of the studies of IGF-1 in ANS dysfunction in Rett syndrome with epilepsy, the potential role of BDNF and IGF-1 in regulating ANS function and cerebral autoregulation in patients with epilepsy has not been reported. Studies have shown trophic effects of BDNF on sympathetic neurons, and BDNF increases brainstem parasympathetic neuron excitability (11, 41). BDNF+/− mice exhibit an elevated heart rate, suggesting that BDNF is involved in regulating sympathetic and/or parasympathetic inputs to the heart (11). In the present study, patient with epilepsy who have lower serum levels of BDNF showed impaired adrenergic and sudomotor function, and higher total scores of autonomic dysfunction, particularly in patients who have focal motor seizures. In addition, patients with reduced serum levels of IGF-1 showed changes in cardiovagal function that may be related to a longer duration of epilepsy, higher seizure frequency, and temporal lobe epilepsy. One measured parameter of cerebral autoregulation (ASI) was significantly correlated with serum levels of BDNF, while another cerebral autoregulation parameter (BHI) was significantly correlated with serum levels of IGF-1. In hypertensive IGF-1-deficient mice, the protective function of cerebral autoregulation was markedly disrupted, which potentially exacerbated cerebromicrovascular injury and neuroinflammation (42). Our results indicate a possible role for BDNF and IGF-1 in regulation ANS functions and cerebral autoregulation in patients with epilepsy.

The ANS is considerably influenced by the CNS and neuroendocrine systems involved in stress response, including epileptic seizures (43). The ANS is regulated by activity in neural circuits of several different stress-responsive brain regions, such as the prefrontal cortex, amygdala, and hippocampus, and by the hypothalamic-pituitary-adrenal (HPA) neuroendocrine system (43, 44). In the brain, BDNF is active in the hippocampus, cortex, and basal forebrain, and also regulates the activity of neurons in the hypothalamus (11). In addition, the IGF-1 receptor is predominantly expressed in the cortex, subventricular zone-olfactory bulb, hippocampus, and hypothalamus (45). Therefore, our results suggest that severe and long-term epilepsy, particularly temporal lobe epilepsy, may perturb BDNF and IGF-1 signaling in central autonomic system and disrupt limbic circuitry and HPA axis regulation, which contribute to the dysfunction of the ANS and the impaired cerebral autoregulation in patients with epilepsy. Perhaps autonomic dysfunction and impaired cerebral autoregulation in patients with epilepsy may also increase the risk of SUDEP.

We are aware that AED therapy may have effects on serum levels of BDNF and IGF-1. Based on animal and human studies (46–50), changes in the expression of BDNF and IGF-1 in brain tissue or circulation have been reported with phenytoin, lamotrigine, valproate, carbamazepine, oxcarbazepine, topiramate, and levetiracetam treatment. However, results from these studies have been inconsistent and the significance of the results was controversial in different animal models and patient groups. In our study, based on a correlation analysis, we found that patients prescribed phenobarbital had higher serum levels of BDNF (*p* = 0.029) and lower levels of IGF-1 (*p* = 0.010) than did patients not taking phenobarbital. Patients prescribed valproate had higher serum levels of BDNF than did patients not taking valproate (*p* = 0.015). However, most of the patients were diagnosed with drug-resistant epilepsy, and received complex and multiple AED therapies (80.7%). Thus, we cannot make any firm conclusions regarding the effect of AEDs on serum levels of BDNF and IGF-1. The influence of AEDs on the expression of BDNF and IGF-1 needs further study in a larger number of patients.

## Conclusion

The present study provides novel insights into clinical observations regarding epilepsy by showing that reduced serum levels of BDNF and IGF-1, autonomic dysfunction, and impaired cerebral autoregulation are present in patients with focal epilepsy. Long-term epilepsy and severe epilepsy, particularly temporal lobe epilepsy, may perturb BDNF and IGF-1 signaling, which contribute to autonomic dysfunction and impaired cerebral autoregulation in patients with epilepsy. These results are important to our understanding of the pathogenesis of ANS dysfunction in patients with epilepsy and have implications for therapeutic interventions targeting BDNF and IGF-1 signaling to correct central autonomic dysfunction and impairment of cerebral autoregulation, particularly in older patients and individuals at high risk for SUDEP.

## Ethics statement

Ethical approval for this study was provided by the Chang Gung Medical Foundation Institutional Review Board, and informed written consent was obtained from all subjects.

## Author contributions

S-FC and Y-CC participated in the study concept and design, performed the study and drafted the manuscript. S-BJ, N-CC, C-RH, M-HT, T-YT, C-CC, and W-CT contributed to the acquisition of data and interpretation of data. H-YC and S-FC performed statistical analysis. Y-CC helped draft the work and revised it critically for important intellectual content.

### Conflict of interest statement

The authors declare that the research was conducted in the absence of any commercial or financial relationships that could be construed as a potential conflict of interest. The handling editor declared a shared affiliation, though no other collaboration, with the authors.
